# Federated Learning of Electronic Health Records to Improve Mortality Prediction in Hospitalized Patients With COVID-19: Machine Learning Approach

**DOI:** 10.2196/24207

**Published:** 2021-01-27

**Authors:** Akhil Vaid, Suraj K Jaladanki, Jie Xu, Shelly Teng, Arvind Kumar, Samuel Lee, Sulaiman Somani, Ishan Paranjpe, Jessica K De Freitas, Tingyi Wanyan, Kipp W Johnson, Mesude Bicak, Eyal Klang, Young Joon Kwon, Anthony Costa, Shan Zhao, Riccardo Miotto, Alexander W Charney, Erwin Böttinger, Zahi A Fayad, Girish N Nadkarni, Fei Wang, Benjamin S Glicksberg

**Affiliations:** 1 The Hasso Plattner Institute for Digital Health at Mount Sinai Icahn School of Medicine at Mount Sinai New York, NY United States; 2 The Mount Sinai Clinical Intelligence Center New York, NY United States; 3 Department of Population Health Sciences Weill Cornell Medicine New York, NY United States; 4 Department of Genetics and Genomic Sciences Icahn School of Medicine at Mount Sinai New York, NY United States; 5 Intelligent System Engineering Indiana University Bloomington, IN United States; 6 School of Information University of Texas Austin Austin, TX United States; 7 Institute for Healthcare Delivery Science Department of Population Health Science and Policy Icahn School of Medicine at Mount Sinai New York, NY United States; 8 Department of Neurological Surgery Icahn School of Medicine at Mount Sinai New York, NY United States; 9 Department of Anesthesiology Perioperative and Pain Medicine Icahn School of Medicine at Mount Sinai New York, NY United States; 10 The Pamela Sklar Division of Psychiatric Genomics Icahn School of Medicine at Mount Sinai New York, NY United States; 11 Department of Psychiatry Icahn School of Medicine at Mount Sinai New York, NY United States; 12 Digital Health Center Hasso Plattner Institute University of Potsdam Potsdam Germany; 13 The BioMedical Engineering and Imaging Institute Icahn School of Medicine at Mount Sinai New York, NY United States; 14 Department of Radiology Icahn School of Medicine at Mount Sinai New York, NY United States; 15 Department of Medicine Icahn School of Medicine at Mount Sinai New York, NY United States; 16 The Charles Bronfman Institute for Personalized Medicine Icahn School of Medicine at Mount Sinai New York, NY United States

**Keywords:** federated learning, COVID-19, machine learning, electronic health records

## Abstract

**Background:**

Machine learning models require large datasets that may be siloed across different health care institutions. Machine learning studies that focus on COVID-19 have been limited to single-hospital data, which limits model generalizability.

**Objective:**

We aimed to use federated learning, a machine learning technique that avoids locally aggregating raw clinical data across multiple institutions, to predict mortality in hospitalized patients with COVID-19 within 7 days.

**Methods:**

Patient data were collected from the electronic health records of 5 hospitals within the Mount Sinai Health System. Logistic regression with L1 regularization/least absolute shrinkage and selection operator (LASSO) and multilayer perceptron (MLP) models were trained by using local data at each site. We developed a pooled model with combined data from all 5 sites, and a federated model that only shared parameters with a central aggregator.

**Results:**

The LASSO_federated_ model outperformed the LASSO_local_ model at 3 hospitals, and the MLP_federated_ model performed better than the MLP_local_ model at all 5 hospitals, as determined by the area under the receiver operating characteristic curve. The LASSO_pooled_ model outperformed the LASSO_federated_ model at all hospitals, and the MLP_federated_ model outperformed the MLP_pooled_ model at 2 hospitals.

**Conclusions:**

The federated learning of COVID-19 electronic health record data shows promise in developing robust predictive models without compromising patient privacy.

## Introduction

COVID-19 has led to over 1 million deaths worldwide and other devastating outcomes [1]. The accurate prediction of COVID-19 outcomes requires data from large, diverse patient populations; however, pertinent data are siloed. Although many studies have produced significant findings for COVID-19 outcomes by using single-hospital data, larger representation from additional populations is needed for generalizability, especially for the generalizability of machine learning applications [2-11]. Large-scale initiatives have been combining local meta-analysis and statistics data derived from several hospitals, but this framework does not provide information on patient trajectories and does not allow for the joint modeling of data for predictive analysis [12,13].

In light of patient privacy, federated learning has emerged as a promising strategy, particularly in the context of COVID-19 [14]. Federated learning allows for the decentralized refinement of independently built machine learning models via the iterative exchange of model parameters with a central aggregator, without sharing raw data. Several studies have assessed machine learning models that use federated learning in the context of COVID-19 and have shown promise. Kumar et al. built a blockchain-based federated learning schema and achieved enhanced sensitivity for detecting COVID-19 from lung computed tomography scans [15]. Additionally, Xu et al. used deep learning to identify COVID-19 from computed tomography scans from multiple hospitals in China, and found that models built on data from hospitals in 1 region did not generalize well to hospitals in other regions. However, they were able to achieve considerable performance improvements when they used a federated learning approach [16]. A more detailed background on COVID-19, machine learning in the context of COVID-19, challenges for multi-institutional collaborations, and federated learning can be found in Multimedia Appendices 1-8.

Although federated learning approaches have been proposed, to our knowledge there have been no published studies that implement, or assess the utility of, federated learning to predict key COVID-19 outcomes from electronic health record (EHR) data [17]. The aim of this study was not to compare the performance of various classifiers in a federated learning environment, but to assess if a federated learning strategy could outperform locally trained models that use 2 common modeling techniques in the context of COVID-19. We are the first to build federated learning models that use EHR data to predict mortality in patients diagnosed with COVID-19 within 7 days of hospital admission.

## Methods

### Clinical Data Source and Study Population

**Figure 1:**
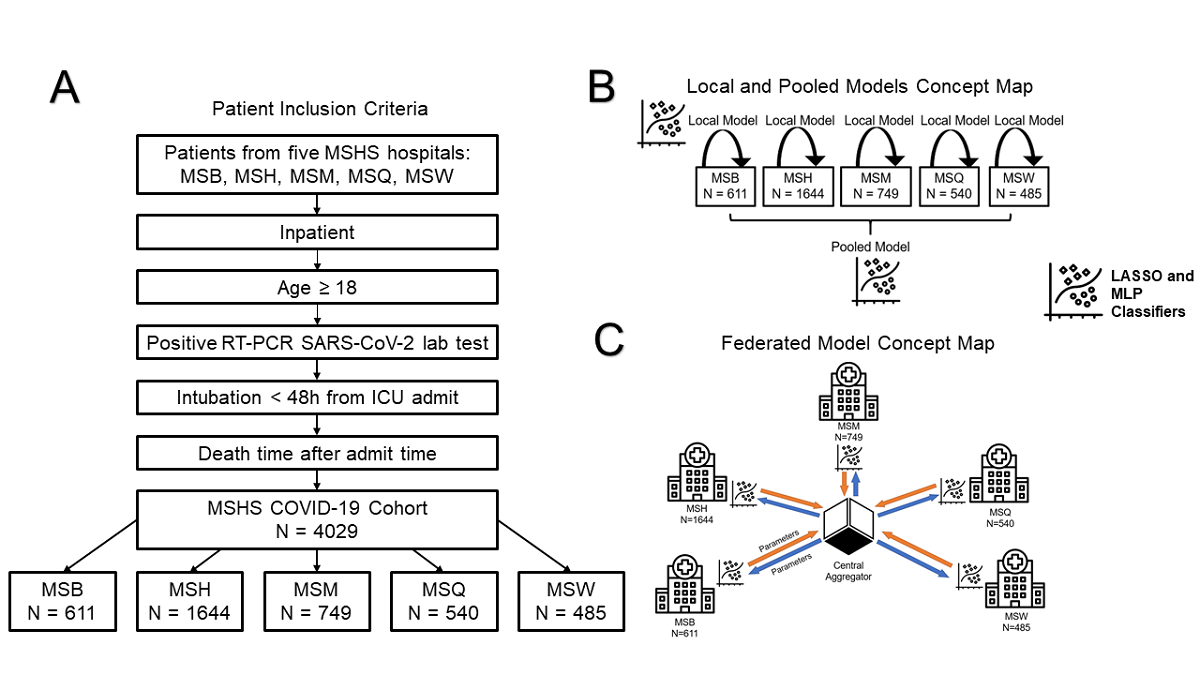
Study design and model workflow. (A) Criteria for patient inclusion in this study. (B) An overview of the local and pooled models. Local models only used data from the site itself, whereas pooled models incorporated data from all sites. Both the local and pooled MLP and LASSO models were used. (C) An overview of the federated model. Parameters from a central aggregator are shared with each site, and sites do not have direct access to clinical data from other sites. After the models are locally trained at a site, parameters with and without added noise are sent back to the central aggregator to update federated model parameters. Federated LASSO and MLP models were used. LASSO: least absolute shrinkage and selection operator; MLP: multilayer perceptron; MSB: Mount Sinai Brooklyn; MSH: Mount Sinai Hospital; MSM: Mount Sinai Morningside; MSQ: Mount Sinai Queens; MSW: Mount Sinai West.

Data from patients who tested positive for COVID-19 (N=4029) were derived from the EHRs of 5 Mount Sinai Health System (MSHS) hospitals in New York City. Study inclusion criteria are shown in Figure 1. Further details, as well as cross-hospital demographic and clinical comparisons, are in Multimedia Appendices 1-8.

### Study Design

We performed multiple experiments, as outlined in Figure 1. First, we developed classifiers that used, and were tested on, local data from each hospital separately. Second, we built a federated learning model by averaging the model parameters of each individual hospital. Third, we combined all individual hospital data into a superset to develop a pooled model that represented an ideal framework.

Study data included the demographics, past medical history, vital signs, lab test results, and outcomes of all patients (Table 1, Table S1 in Multimedia Appendix 2). Due to the varying prevalence of COVID-19 across hospitals, we assessed multiple class balancing techniques (Table S2 in Multimedia Appendix 3). To simulate federated learning in practice, we also performed experiments with the addition of Gaussian noise (Multimedia Appendix 7). To promote replicability, we used the TRIPOD (Transparent Reporting of a Multivariable Prediction Model for Individual Prognosis or Diagnosis) guidelines (Table S3 in Multimedia Appendix 4) and released our code under a general public license (Multimedia Appendices 1-8).

**Table 1:** Demographic characteristics of all hospitalized patients with COVID-19 included in this study (N=4029)^a^.

Characteristic		Mount Sinai Brooklyn	Mount Sinai Hospital	Mount Sinai Morningside	Mount Sinai Queens	Mount Sinai West	*P* value
Number of patients, n		611	1644	749	540	485	—^b^
**Gender, n (%)**							
	Male	338 (55.3)	951 (57.8)	411 (54.9)	344 (63.7)	257 (53.0)	.004
	Female	273 (44.7)	693 (42.2)	338 (45.1)	196 (36.3)	228 (47.0)	.004
Age (years), median (IQR)		72.5 (63.6-82.7)	63.3 (51.3-73.2)	69.8 (57.4-80.3)	68.1 (57.1-78.8)	66.3 (52.5-77.6)	<.001
**Ethnicity, n (%)**							
	Hispanic	21 (3.4)	460 (28.0)	259 (34.6)	198 (36.7)	111 (22.9)	<.001
	Non-Hispanic	416 (68.1)	892 (54.3)	452 (60.3)	287 (53.1)	349 (72.0)	<.001
	Unknown	174 (28.5)	292 (17.8)	38 (5.1)	55 (10.2)	25 (5.2)	<.001
**Race, n (%)**							
	Asian	13 (2.1)	83 (5.0)	16 (2.1)	56 (10.4)	27 (5.6)	<.001
	Black/African American	323 (52.9)	388 (23.6)	266 (35.5)	64 (11.9)	109 (22.5)	<.001
	Other	54 (8.8)	705 (42.9)	343 (45.8)	288 (53.3)	164 (33.8)	<.001
	Unknown	27 (4.4)	87 (5.3)	25 (3.3)	14 (2.6)	14 (2.9)	<.001
	White	194 (31.8)	381 (23.2)	99 (13.2)	118 (21.9)	171 (35.3)	<.001
**Past medical history, n (%)**							
	Acute myocardial infarction	14 (2.3)	16 (1.0)	—	15 (2.8)	7 (1.4)	.006
	Acute respiratory distress syndrome	—	28 (1.7)	—	—	—	<.001
	Acute venous thromboembolism	—	11 (0.7)	—	—	—	.74
	Asthma	—	100 (6.1)	39 (5.2)	19 (3.5)	27 (5.6)	<.001
	Atrial fibrillation	23 (3.8)	113 (6.9)	44 (5.9)	49 (9.1)	28 (5.8)	.005
	Cancer	22 (3.6)	190 (11.6)	47 (6.3)	21 (3.9)	41 (8.5)	<.001
	Chronic kidney disease	46 (7.5)	208 (12.7)	75 (10.0)	81 (15.0)	33 (6.8)	<.001
	Chronic obstructive pulmonary disease	11 (1.8)	64 (3.9)	31 (4.1)	28 (5.2)	19 (3.9)	.04
	Chronic viral hepatitis	—	17 (1.0)	14 (1.9)	—	—	.02
	Coronary artery disease	56 (9.2)	168 (10.2)	92 (12.3)	82 (15.2)	51 (10.5)	.008
	Diabetes mellitus	93 (15.2)	351 (21.4)	165 (22.0)	154 (28.5)	76 (15.7)	<.001
	Heart failure	36 (5.9)	110 (6.7)	61 (8.1)	43 (8.0)	30 (6.2)	.38
	Human immunodeficiency virus	—	32 (1.9)	11 (1.5)	—	14 (2.9)	.001
	Hypertension	112 (18.3)	549 (33.4)	249 (33.2)	225 (41.7)	139 (28.7)	<.001
	Intracerebral hemorrhage	—	—	—	—	—	.24
	Liver disease	—	53 (3.2)	15 (2.0)	15 (2.8)	—	<.001
	Obesity	—	176 (10.7)	74 (9.9)	38 (7.0)	29 (6.0)	<.001
	Obstructive sleep apnea	—	54 (3.3)	15 (2.0)	—	—	<.001
	Stroke	—	24 (1.5)	—	—	—	.054
Mortality within 7 days, n (%)		148 (24.2)	118 (7.2)	93 (12.4)	124 (23.0)	27 (5.6)	<.001

^a^Interhospital comparisons for categorical data were assessed with Chi-square tests. Numerical data were assessed with Kruskal-Wallis tests, and Bonferroni-adjusted *P* values were reported. Values relating to <10 patients per field were not provided to protect patient privacy (--).

^b^Not available.

### Model Development and Selection

The primary outcome was mortality within 7 days of admission. We generated 2 baseline conventional predictive models—a multilayer perceptron (MLP) model and a logistic regression with L1-regularization or least absolute shrinkage and selection operator (LASSO) model. To maintain consistency and enable direct comparisons, each MLP model was built with the same architecture. We provide more information on model architecture and tuning in Multimedia Appendix 5. MLP and LASSO models were fit on all 5 hospitals.

Our primary model of interest was a federated learning model. Training was performed at different sites, and parameters were sent to a central location (Figure 1). A central aggregator was used to initialize the federated model with random parameters. This model was sent to each site and trained for 1 epoch. Afterward, model parameters were sent back to the central aggregator, which is where federated averaging was performed. Updated parameters from the central aggregator were then sent back to each site. This cycle was repeated for multiple epochs. Federated averaging scales the parameters of each site according to the number of available data points and sums all parameters by layer. Through this technique, federated models did not receive any raw data.

### Experimental Evaluation

All models were trained and evaluated by using 490-fold bootstrapping. Each experiment had a 70%-30% training-testing data split and was initialized with a unique random seed. We used the models’ probability scores to calculate average areas under the receiver operating characteristic curve (AUROCs) across 490 iterations.

## Results

### Intercohort Comparisons

EHR data consisted of patient demographics, past medical history, vitals, and lab test results (Table 1, Table S1 in Multimedia Appendix 2). After performing Bonferroni correction, we found significant differences in the proportions of outcomes across hospitals, specifically mortality within 7 days (Table 1). There were also significant differences in gender, age, ethnicity, race, and the majority of key clinical features (Table S1 in Multimedia Appendix 2).

### Classifier Training and Performance

**Figure 2:**
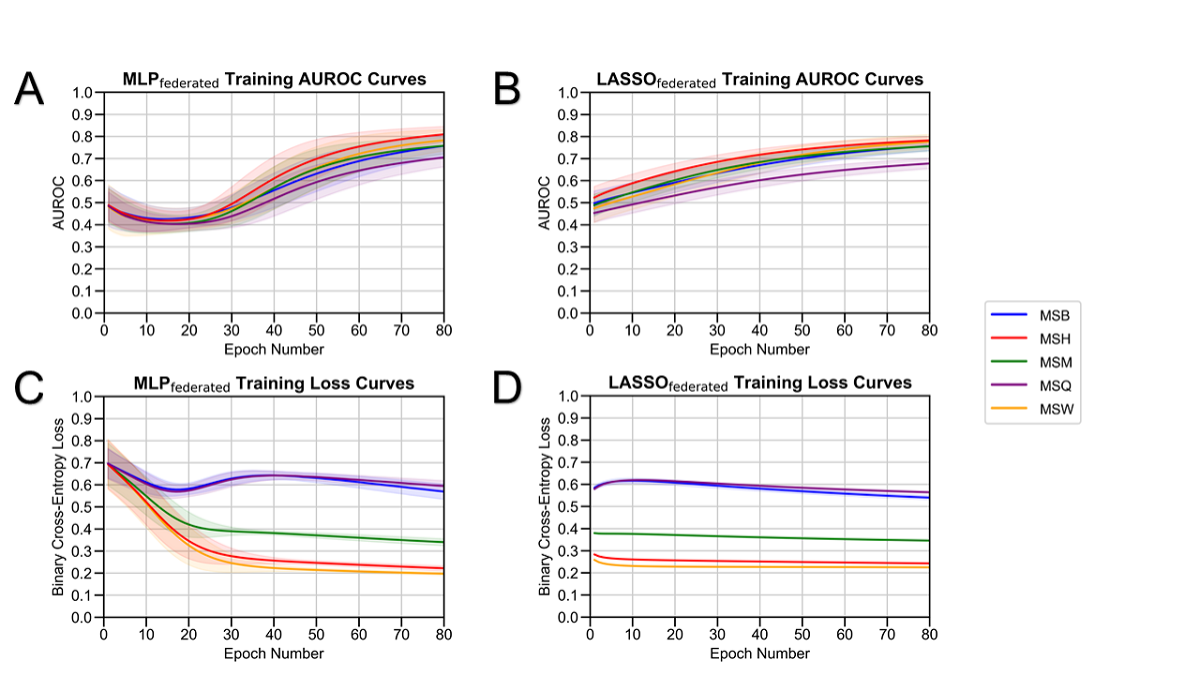
Federated model training. The performance of (A) federated MLP and (B) federated LASSO models, as measured by AUROCs versus the number of training epochs. The binary cross-entropy loss of (C) federated MLP and (D) federated LASSO models versus the number of training epochs. AUROC: area under the receiver operating characteristic curve; LASSO: least absolute shrinkage and selection operator; MLP: multilayer perceptron; MSB: Mount Sinai Brooklyn; MSH: Mount Sinai Hospital; MSM: Mount Sinai Morningside; MSQ: Mount Sinai Queens; MSW: Mount Sinai West.

LASSO and MLP models were trained on data from each of the 5 MSHS hospitals separately (ie, local models), data from a combined dataset (ie, pooled models), and data from a federated learning framework (ie, federated models). All 3 training strategies for both models were evaluated for all sites (Figure 1). Training curves and AUROC curves versus the epoch number demonstrate that federated models improve performance after increased passes of training data (Figure 2). The results for model optimization (Figure S2 in Multimedia Appendix 8) and class balancing experiments (Table S2 in Multimedia Appendix 3) can be found in Multimedia Appendices 1-8. The final model hyperparameters are listed in Table S4 in Multimedia Appendix 5.

### Learning Framework Comparisons

**Figure 3:**
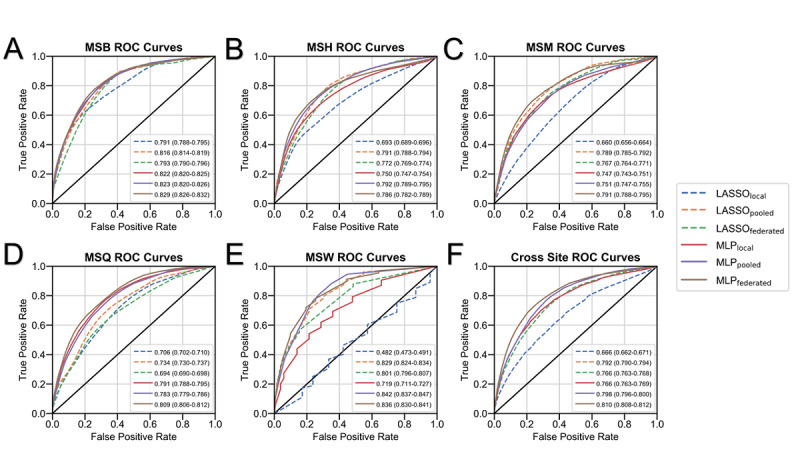
Model performance by site. The performance of all models (ie, local LASSO, pooled LASSO, federated LASSO, local MLP, pooled MLP, and federated [no noise] MLP models) based on areas under the ROC curve at (A) MSB (n=611), (B) MSW (n=485), (C) MSM (n=749), (D) MSH (n=1644), and (E) MSQ (n=540). Average areas under the ROC curve with 95% confidence intervals (ie, after the 70%-30% training-testing data split over 490 experiments) are shown. (F) The average performance of each model across all 5 sites. LASSO: least absolute shrinkage and selection operator; MLP: multilayer perceptron; MSB: Mount Sinai Brooklyn; MSH: Mount Sinai Hospital; MSM: Mount Sinai Morningside; MSQ: Mount Sinai Queens; MSW: Mount Sinai West; ROC: receiver operating characteristic.

The performance of all LASSO and MLP models (ie, local, pooled, and federated models) was assessed at each site (Table 2, Figure 3). The LASSO_federated_ model outperformed the LASSO_local_ model at all hospitals except the Mount Sinai Brooklyn and Mount Sinai Queens hospitals; the LASSO_federated_ model achieved AUROCs that ranged from 0.694 (95% CI 0.690-0.698) to 0.801 (95% CI 0.796-0.807). The LASSO_pooled_ model outperformed the LASSO_federated_ model at all hospitals; the LASSO_pooled_ model achieved AUROCs that ranged from 0.734 (95% CI 0.730-0.737) to 0.829 (95% CI 0.824-0.834).

The MLP_federated_ model outperformed the MLP_local_ model at all hospitals; the MLP_federated_ model achieved AUCROCs that varied from 0.786 (95% CI 0.782-0.789) to 0.836 (95% CI 0.830-0.841), while the MLP_local_ model achieved AUROCs that ranged from 0.719 (95% CI 0.711-0.727) to 0.822 (95% CI 0.820-0.825). The MLP_federated_ model outperformed the MLP_pooled_ model at the Mount Sinai Morningside and Mount Sinai Queens hospitals; the MLP_pooled_ model achieved AUROCs that ranged from 0.751 (95% CI 0.747-0.755) to 0.842 (95% CI 0.837-0.847).

**Table 2:** Performance of the local, pooled, and federated LASSO^a^ and MLP^b^ models at each site, based on AUROCs^c^ with 95% confidence intervals.

Model		Mount Sinai Brooklyn (n=611), AUROC (95% CI)	Mount Sinai Hospital (n=1644), AUROC (95% CI)	Mount Sinai Morningside (n=749), AUROC (95% CI)	Mount Sinai Queens (n=540), AUROC (95% CI)	Mount Sinai West (n=485), AUROC (95% CI)
**LASSO model**						
	Local	0.791 (0.788-0.795)	0.693 (0.689-0.696)	0.66 (0.656-0.664)	0.706 (0.702-0.710)	0.482 (0.473-0.491)
	Pooled	0.816 (0.814-0.819)	0.791 (0.788-0.794)	0.789 (0.785-0.792)	0.734 (0.730-0.737)	0.829 (0.824-0.834)
	Federated	0.793 (0.790-0.796)	0.772 (0.769-0.774)	0.767 (0.764-0.771)	0.694 (0.690-0.698)	0.801 (0.796-0.807)
**MLP model**						
	Local	0.822 (0.820-0.825)	0.750 (0.747-0.754)	0.747 (0.743-0.751)	0.791 (0.788 - 0.795)	0.719 (0.711-0.727)
	Pooled	0.823 (0.820-0.826)	0.792 (0.789-0.795)	0.751 (0.747-0.755)	0.783 (0.779-0.786)	0.842 (0.837-0.847)
	Federated (no noise	0.829 (0.826-0.832)	0.786 (0.782-0.789)	0.791 (0.788-0.795)	0.809 (0.806-0.812)	0.836 (0.83-0.841)

^a^LASSO: least absolute shrinkage and selection operator.

^b^MLP: multilayer perceptron.

^c^AUROC: area under the receiver operating characteristic curve.

## Discussion

This is the first study to evaluate the efficacy of applying federated learning to the prediction mortality in patients with COVID-19. EHR data from 5 hospitals were used to represent demonstrative use cases. By using disparate patient characteristics from each hospital after performing multiple-hypothesis correction in terms of demographics, outcomes, sample size, and lab values, this study was able to reflect a real-world scenario, in which federated learning could be used for diverse patient populations.

The primary findings of this study show that the MLP_federated_ and LASSO_federated_ models outperformed their respective local models at most hospitals. Differences in MLP model performance may have been attributed to the experimental condition, wherein the same underlying architecture was used for all MLP models. Although this framework allowed for consistency in learning strategy comparisons, it may have led to the improper tuning of pooled models. Collectively, our results show the potential of federated learning in overcoming the drawbacks of fragmented, case-specific local models.

Our study shows scenarios in which federated models should either be approached with caution or favored. The Mount Sinai Queens hospital was the only hospital where the LASSO_federated_ model performed worse than the LASSO_local_ model, with a difference of 0.012 in AUROC values. This may have been attributed to the hospital having a smaller sample size (n=540) and higher mortality prevalence (23%) than the other sites. However, at the Mount Sinai West hospital, the LASSO_local_ model severely underperformed compared to the LASSO_federated_ model, with an AUROC difference of 0.319. The Mount Sinai West hospital had the lowest sample size (n=485) and the lowest COVID-19 mortality prevalence (5.6%) compared to all hospitals. This finding emphasizes the benefit of using federated learning for sites with small sample sizes and large class imbalances.

We noted a few limitations in our study. First, data collection was limited to MSHS hospitals. This may limit model generalizability to hospitals in other regions. Second, this study focused on applying federated learning to the prediction of outcomes based on patient EHR data as proof of principle, rather than creating an operational framework for immediate deployment. As such, there are various aspects of the federated learning process that this study does not address, such as load balancing, convergence, and scaling. Third, our models only included clinical data. The models can be enhanced by incorporating other modalities. Fourth, we only implemented 2 widely used classifiers within this framework, but other algorithms may perform better. Finally, although identical MLP architectures were used across all learning strategies for direct comparisons, these architectures could have been further optimized. Future studies should focus on model accessibility and the expansion analysis of federated models to improve scalability, understand feature importance, and integrate additional data modalities.

## Appendix

Multimedia Appendix 1 Supplementary materials.

Multimedia Appendix 2 Table S1. Clinical characteristics of hospitalized patients with COVID-19 at baseline. The clinical characteristics of all patients (N=4029) included in this study, including vital signs, metabolic markers, liver function, inflammatory markers, and hematological markers. All laboratory data was obtained within 36 hours of admission. Interhospital comparisons for categorical data were assessed with Chi-square tests. Numerical data were assessed Kruskal-Wallis tests. Bonferroni-adjusted *P* values are reported. Values relating to <10 patients per field are not provided to protect patient privacy.

Multimedia Appendix 3 Table S2. Effects of class balancing techniques on local MLP models based on AUROCs and AUPRCs. Local MLP model performance, as measured by the AUROCs and AUPRCs of the 3 class balancing techniques (ie, static class weights, proportional class weights, and 1:1 undersampling) and unbalanced data for all 5 sites after training for 80 epochs. The outcome of interest, mortality percentage within seven days, is provided for each site. AUPRC: area under the precision-recall curve; AUROC: area under the receiver operating characteristic curve; MLP: multilayer perceptron.

Multimedia Appendix 4 Table S3. Study data as reported using Transparent Reporting of a Multivariable Prediction Model for Individual Prognosis or Diagnosis (TRIPOD) guidelines.

Multimedia Appendix 5 Table S4. Final model hyperparameters. The LASSO and MLP model hyperparameters used at all sites for all variations (ie, local, pooled, and federated models), after optimization. LASSO: least absolute shrinkage and selection operator; MLP: multilayer perceptron.

Multimedia Appendix 6 Table S5. Model performance metrics across sites. The performance of all LASSO and MLP models (ie, local, pooled, and federated models), as measured by AUROCs, AUPRCs, accuracy, sensitivity, specificity, and F1 score, with 95% confidence intervals. AUPRC: area under the precision recall curve; AUROC: area under the receiver operating characteristic curve; LASSO: least absolute shrinkage and selection operator; MLP: multilayer perceptron.

Multimedia Appendix 7 Effect of noise on federated MLP model performance by site. The performance of federated MLP models without noise and federated MLP models with Gaussian noise, as determined by AUROCs, was assessed at (A) MSB (n=611) (B) MSW (n=485), (C) MSM (n=749), (D) MSH (n=1644), and (E) MSQ (n=540) after 70-30 train-test split over 490 experiments. (F) The average performance of both federated MLP models across all 5 sites. AUROC: area under the receiver operating characteristic curve; MLP: multilayer perceptron; MSB: Mount Sinai Brooklyn; MSH: Mount Sinai Hospital; MSM: Mount Sinai Morningside; MSQ: Mount Sinai Queens; MSW: Mount Sinai West.

Multimedia Appendix 8 Effect of noise on federated MLP model training. The performance of federated MLP models without noise and federated MLP models with Gaussian noise was evaluated by using (A) AUROCs and (B) binary cross-entropy loss versus the number of training epochs. The performance of federated MLP models with Gaussian noise was assessed with (C) AUROCs and (D) binary cross-entropy loss at all 5 sites. The averages after the 70%-30% training-testing data split over 490 experiments were used for all plots. AUROC: area under the receiving-operating characteristic curve; MLP: multilayer perceptron; MSB: Mount Sinai Brooklyn; MSH: Mount Sinai Hospital; MSM: Mount Sinai Morningside; MSQ: Mount Sinai Queens; MSW: Mount Sinai West.

## Abbreviations

AUROC: area under the receiver operating characteristic curve
EHR: electronic health record
LASSO: least absolute shrinkage and selection operator
MLP: multilayer perceptron
MSHS: Mount Sinai Health System
TRIPOD: Transparent Reporting of a Multivariable Prediction Model for Individual Prognosis or Diagnosis
