# Supplementary material for: Federated Learning of Electronic Health Records to Improve Mortality Prediction in Hospitalized Patients With COVID-19: Machine Learning Approach
Source: JMIR Med Inform. 2021 Jan 27;9(1):e24207. doi: 10.2196/24207 (PMC7842859; doi:10.2196/24207)
Supplement: Multimedia Appendix 1 [file medinform_v9i1e24207_app1.doc]

## SUPPLEMENTARY MATERIALS

### Supplementary Introduction

#### COVID-19 Background

Cases of the novel Coronavirus SARS-CoV-2 (COVID-19) were first reported in December 2019 in Wuhan, China [1]. Since then, the virus has quickly spread and infected over 41 million individuals, killing over one million [2]. In March 2020, New York City became the epicenter of this pandemic in the United States. By August, New York City has faced over 20,000 deaths among over 200,000 infected individuals [2]. Consequently, New York healthcare systems quickly became overburdened by increased clinical demand, leading to challenges with personal protective equipment availability and hospital admissions capacity [3].

#### Machine Learning in COVID-19

The swift rise in COVID-19 case count has suggested that the use of machine learning to handle this large amount of information may be effective for prediction of COVID-19 related outcomes, such as mortality, for patient triage, and surveillance, among others [4]. There are several studies that demonstrate this promise. Yan et al. identified three biomarkers that were able to predict mortality in patients more than ten days in advance with over 90% accuracy [5]. Using a smartphone-based app, Menni et al. identified key self-reported clinical features that correlated with a positive COVID-19 test result [6]. Liang et al. built a machine learning classification tool to guide patient triage at admission for those at greatest risk of severe illness [7]. While the results of these studies show promise, many of them were built on small sample sizes and patients specific to one region which may not generalize to the entire population.

#### Barriers in the Context of Multi-Institutional EHR Projects

Creating generalizable machine learning models for use in healthcare often requires a substantial amount of diverse data from many hospital systems. In an ideal world, patient data from all hospital systems would be in a centralized and harmonized repository to build models. In modern healthcare, however, this centralized strategy is difficult to implement in practice. There are relevant challenges as data generally originates from various locations, including hospitals, clinics, pharmacies, payors, and personal devices, all of which are fragmented and private. Accordingly, even integrating data from health systems themselves is challenging due to issues of patient privacy and safety, as well as regulation and infrastructure. For example, a single point-of-failure in the security framework can result in the disastrous loss of privacy for collections of private patient data [8]. Given these challenges in multi-institutional data sharing and privacy preservation, a distributed approach to work with health data from multiple sites could be more fitting.

#### Background on Federated Learning

Federated learning is where localized data at individual sites are used to train separate models. Model parameters are subsequently aggregated to produce the federated model which averages the weights and biases from contributing sites [8,9]. However, federated learning is not without its issues: health data is typically non-identically, independently distributed (non-IID) which can present with severe class imbalances [10]. These limitations, which are common in real-world healthcare settings, may negatively affect the performance of certain federated learning models [11]. Studies looking at the use of federated learning with distributed electronic health record data found that these algorithms were comparable to centralized learning and could achieve similar prediction accuracy [10-12]. Choudhury et al. were able to enhance the prediction of adverse drug reactions using federated learning that approximated performance of centralized learning and outperformed locally-derived models [12]. Li et al. demonstrated that a federated learning strategy for deep learning of fMRI data boosted performance of neurological-related image analysis for identifying relevant biomarkers [13].

### Supplementary Methods and Materials

#### Study Inclusion Criteria

We included adult patients, aged 18 and over, with laboratory-confirmed COVID-19 infection. Patients were admitted between March 15^th^ and May 22^nd^ 2020, to any of five MSHS hospitals: Mount Sinai Brooklyn (MSB), Mount Sinai Hospital (MSH), Mount Sinai Morningside (MSM), Mount Sinai Queens (MSQ), or Mount Sinai West (MSW). COVID-19 results were confirmed with a reverse transcriptase polymerase chain reaction (RT-PCR) assay of a nasopharyngeal swab. Patients needed to receive a positive COVID-19 laboratory result within 48 hours of admission to be included in the study and be in the hospital for at least seven days or successfully discharged or died within this timeframe. The requirement for having at least seven days of data is to protect against immortal time bias.

#### Study Data

Demographic data included age, gender, reported race, and ethnicity. Race was collated into categories based on 2010 US census race categories: American Indian or Alaskan Native, Asian, Black or African-American, Other, Native Hawaiian or Other Pacific Islander, Unknown, and White [14]. Patients of Native American and Pacific Islander descent were further collapsed into ‘Other’ as their count was less than ten patients. Ethnicity was collapsed into three categories: Hispanic/Latino, Non-Hispanic/Latino, and Unknown. Using ICD (International Classification of Diseases-9/10-Clinical Modification (ICD-9/10-CM)) codes, we extracted patient’s past medical history to examine pre-existing conditions documented to have increased incidence in hospitalized COVID-19 patients: acute respiratory distress syndrome (ARDS), acute venous thromboembolism, acute myocardial infarction, asthma, atrial fibrillation, cancer, chronic kidney disease (CKD), chronic obstructive pulmonary disease (COPD), chronic viral hepatitis, coronary artery disease (CAD), diabetes mellitus, heart failure, human immunodeficiency virus (HIV), hypertension, intracerebral hemorrhage, obstructive sleep apnea, liver disease, and stroke [15-20]. If a hospital had fewer than ten patients who had a relevant past medical condition, that group was left blank to preserve patient privacy. Additional included clinical data were the first lab value or vital sign obtained within 36 hours of admission. The time window of 36 hours was implemented to allow for the results of these lab tests to be processed and entered into the system. Lab values with less than 30% missingness at each site were utilized, and k-Nearest Neighbors (k=5) was used to impute missing data for these features. Any outliers below 0.5 or above 99.5 percentiles were excluded.

#### Interhospital Cohort Comparisons

After grouping by hospital, medians and interquartile ranges were calculated for continuous data. Statistical significance was defined as Bonferroni-adjusted *P*<0.05 of Kruskal-Wallis or chi-squared tests. Bonferroni correction was conducted by multiplying *P*-values by 62 study-wise tests as sub-groups (gender, race, and ethnicity) were treated as unique hypothesis spaces.

#### Class Balancing

Significant differences in the proportion of mortality were seen across the five Mount Sinai hospitals. As this imbalance can affect model performance, we attempted various class balancing procedures. One method was to use weighted neural networks, where there is fixed weighting of error scores based on class. Large weights are assigned to the minority class (patients dying within seven days), and smaller weights are assigned to the majority class (patients alive after seven days) as errors are more essential to learn from in an imbalanced dataset. We also used static weights of 0.05 for the majority class and 0.95 for the minority class to evaluate model performance, and we calculated model performance using weights equivalent to the proportion of outcome prevalence within a hospital. Next, we performed 1:1 undersampling of the majority class so that there were similar numbers in both classes. Results are shown in Supplementary Table 2.

#### Data Validation

All models were trained and evaluated using 490 fold bootstrapping. Each experiment had a 70-30 training-testing split and was initialized with a unique random seed. We utilized the models’ probability scores to calculate averages of areas under the receiver operating characteristic curve (AUC-ROC) across 490 iterations.

#### Sensitivity and Specificity Confidence Intervals

Confidence intervals for sensitivity and specificity with the cutoff determined by maximum Youden Index were estimated after Box-Cox Transformation as defined by Bantis et al [21]. All model performance metrics are shown in Supplementary Table 5.

#### Differential Privacy

Although our approach to federated learning is more secure than traditional machine learning models as data do not leave their respective location, parameters are still shared. To prevent inferring important properties of patient data from the shared model parameters, e.g., whether or not an individual was a patient at the hospital, differential privacy was further incorporated into the federated model [22-24]. Specifically, the Gaussian mechanism, one of the common choices to achieve differential privacy, was adopted where Gaussian noise was injected to the locally trained model before they were shared to the central location [22]. The effect on model performance was assessed with and without the introduction of noise in Supplemental Figure 1.

#### Promoting Interoperability and Replicability

This article is written following the TRIPOD (Transparent Reporting of a Multivariable Prediction Model for Individual Prognosis or Diagnosis) guidelines, which are further elaborated in Supplementary Table 3 [25]. Furthermore, we release all code used for building the classifier under the GPLv3 license in a public GitHub repository:

<https://github.com/HPIMS/CovidFederatedMortality>

#### Software

All models were built on Python 3.8, PyTorch 1.5, CUDA 10.2, scikit-learn 0.23, pandas 1.0.5, numpy 1.19, and were run on an updated Arch Linux System.

### Supplementary Results/Discussion

#### Model Development

For consistency and to enable direct comparisons, each MLP model was instantiated with three layers. The first 2 layers had ReLU activation, and the output layer had a LogSoftmax activation. The LASSO model had a single layer with Sigmoid activation, with an additional l1 regularization parameter (c=0.1) attached to the loss. Final model hyperparameters are listed in Supplementary Table 4.

#### Model Optimization

Multiple class balancing techniques were assessed to correct differences in mortality rates between the five sites (Supplementary Table 2). Performance was similar between the use of static class weights and proportional class weights as measured by area under the receiver-operating characteristic (AUC-ROC) and area under the precision-recall curve (AUPRC) at all sites. There was a difference of 0.001 in AUC-ROC across all five hospitals between static class weights and proportional class weights. 1:1 undersampling had worse performance than other class balancing techniques as measured by AUC-ROC at three hospitals (MSB,MSH,MSW) and a lower AUPRC at all hospitals except MSM. Class balancing techniques outperformed unbalanced data at all hospitals except MSH in AUC-ROC and AUPRC only at MSM. Although there were trends that class balancing techniques outperformed unbalanced data at some sites, there was no class balancing technique that outperformed unbalanced data in both AUC-ROC and AUPRC in all sites. Therefore, we did not utilize class balancing methods in this study, and there were also fewer barriers to utilizing unbalanced data in federated models.

MLP_federated_ (Figure 2A) and LASSO_federated_ (Figure 2B) models were trained for 80 epochs and their performance on the training dataset of each facility was measured after every epoch. Over 490 experiments, MLP_federated_ models (Figure 2C) began at an average Binary Cross-Entropy loss of approximately 0.7 which decreased to 0.4 at most sites while LASSO_federated_ models (Figure 2D) began at varying rates of Binary Cross-Entropy loss before reaching an average max loss of 0.4 at 80 epochs.

#### Cross-model Comparisons

Although the objective of the study was to not compare the performance of various classifiers to each other, a brief discussion on this topic is warranted. MLP_local_ outperformed LASSO_local_ at all hospitals, and MLP_federated_ surpassed LASSO_federated_ at all hospitals (Table 2). MLP_local_ had AUC-ROCs ranging from 0.719 (95% CI 0.711-0.727) to 0.822 (95% CI 0.820-0.825) while LASSO_local_ had AUC-ROCs from 0.482 (95% CI 0.473-0.491) to 0.791 (95% CI 0.788-0.795). MLP_pooled_ exceeded the performance of LASSO_pooled_ at MSB, MSQ, and MSW and performed worse than LASSO_pooled_ at MSM with a difference of 0.038. MLP_federated_ (without noise) outperformed LASSO_federated_ at all sites, and MLP_federated_ AUC-ROCs varied from 0.786 (95% CI 0.782-0.789) to 0.836 (95% CI 0.830-0.841) while LASSO_federated_ had AUC-ROCs from 0.694 (95% CI 0.690-0.698) to 0.801 (95% CI 0.796-0.807).

Effect of Gaussian Noise

Gaussian noise introduced into MLP_federated_ led to decreased average performance at all sites except MSW where both MLP_federated_ models had AUC-ROCs within confidence intervals of each other (Supplementary Figure 1). AUC-ROCs ranged from 0.786 (95% CI 0.782-0.789) to 0.836 (95% CI 0.830-0.841) in MLP_federated_ without noise and 0.777 (95% CI 0.773-0.780) to 0.831 (95% CI 0.825-0.837) in MLP_federated_ with noise (Supplementary Table 5). Additional performance metrics for all models are described in Supplementary Table 5. Results demonstrate that noise may be inserted to increase data security without compromising federated model performance (Supplemental Figure 2).

### Supplementary References

### Supplementary Figures

Figure 1. Effect of Noise on Federated MLP Performance by Site

Performance of MLP_federated_ without noise and MLP_federated_ with the introduction of Gaussian noise by area under the receiver-operating characteristic (AUC-ROC) was assessed at (A) Mount Sinai Brooklyn (MSB) (n=611) (B) Mount Sinai West (MSW) (n=485), (C) Mount Sinai Morningside (MSM) (n=749), (D) Mount Sinai Hospital (MSH) (n=1644), and (E) Mount Sinai Queens (MSQ) (n=540) after 70-30 train-test split over 490 experiments. Average performance of both federated MLP models across all five sites is presented in (F).

Figure 2. Effect of Noise on Federated MLP Model Training

Performance of MLP_federated_ without noise and MLP_federated_ with the introduction of Gaussian noise was evaluated utilizing (A) area under the receiving-operating characteristic (AUC-ROC) and (B) Binary Cross-Entropy Loss against number of training epochs. Performance of Federated MLP with Gaussian noise was assessed by (C) area under the receiver-operating characteristic (AUC-ROC) and (D) Binary Cross-Entropy Loss at all five sites. Averages after 70-30 train-test split over 490 experiments were used for all plots.

### Supplementary Tables

Supplementary Table 1. Clinical Characteristics of Hospitalized COVID-19 Patients at Baseline.

Clinical characteristics for all patients (n=4029) included in study with breakdown by vital signs, metabolic markers, liver function, inflammatory markers, hematological markers. All laboratory data was obtained within 36 hours of admission. Inter-hospital comparisons for categorical data were assessed with chi-square tests and numerical data using Kruskal-Wallis test with Bonferroni-adjusted *P*-values reported. Values with fewer than ten patients per field are not provided to protect patient privacy.

Supplementary Table 2. Effects of Class Balancing Techniques on MLP LocalAUC-ROC and AUPRC.

MLP_local_ performance as measured by area under the receiver operating-characteristic (AUC-ROC) and area under the precision-recall curve (AUPRC) of three class balancing techniques (static class weights, proportional class weights, and 1:1 undersampling) and unbalanced data for all five sites after training for 80 epochs. The outcome of interest, mortality percentage within seven days, is given for each site.

Supplementary Table 3. TRIPOD Guidelines Report.

Study data as reported using Transparent Reporting of a Multivariable Prediction Model for Individual Prognosis or Diagnosis (TRIPOD) guidelines.

Supplementary Table 4. Final Model Hyper-Parameters.

LASSO and MLP model hyper-parameters utilized at all sites and all variations (local, pooled, federated) after optimization.

Supplementary Table 5. Model Performance Metrics Across Sites.

Performance of all LASSO and MLP models (local, pooled, federated) as measured by area under the receiver operating-characteristic (AUC-ROC), area under the precision recall curve (AUC-PRC), accuracy (ACC), sensitivity (SENS), specificity (SPEC), and F1-score (F1S) with 95% confidence intervals.
