# Supplementary material for: Federated Learning of Electronic Health Records to Improve Mortality Prediction in Hospitalized Patients With COVID-19: Machine Learning Approach
Source: JMIR Med Inform. 2021 Jan 27;9(1):e24207. doi: 10.2196/24207 (PMC7842859; doi:10.2196/24207)
Supplement: Multimedia Appendix 2 [file medinform_v9i1e24207_app2.pdf]

Supplementary Table 1: Clinical Characteristics of Hospitalized COVID Patients at Baseline.

	**Mount Sinai Brooklyn (MSB)**	**Mount Sinai Hospital (MSH)**	**Mount Sinai Morningside (MSM)**	**Mount Sinai Queens (MSQ)**	**Mount Sinai West (MSW)**	**P-Value**	**% Missing**
**(N)**	611	1644	749	540	485		
**Vital Signs at Hospital Admission, median (IQR)**							
Heart Rate, bpm	87.0 [76.0,99.0]	87.0 [76.0,97.0]	87.0 [76.0,98.0]	87.0 [76.0,99.0]	82.0 [73.0,95.0]	0.002	0.02%
Pulse Oximetry, %	97.0 [95.0,98.0]	96.0 [94.0,98.0]	96.0 [94.0,98.0]	96.0 [94.0,97.8]	96.0 [95.0,98.0]	<0.001	0.32%
Respiration Rate, breaths/minute	18.0 [18.0,20.0]	20.0 [18.0,21.0]	19.0 [18.0,21.0]	18.0 [18.0,20.0]	18.0 [17.0,20.0]	<0.001	0.02%
Temperature, F	97.8 [97.1,98.6]	98.6 [98.0,99.8]	98.7 [97.9,99.7]	98.6 [98.0,99.3]	98.5 [97.8,99.2]	<0.001	0.02%
Diastolic Blood Pressure, mm Hg	70.0 [62.0,80.0]	69.0 [61.0,78.0]	73.0 [66.0,82.0]	70.0 [63.0,79.0]	73.0 [64.0,81.0]	<0.001	0.05%
Systolic Blood Pressure, mmHg	128.0 [114.0,141.0]	125.0 [112.0,140.0]	124.0 [111.0,140.0]	126.0 [109.0,140.0]	123.0 [110.0,136.0]	0.011	0.05%
**Admission Laboratory Parameters, median (IQR)**							
**Metabolic Markers**							
Anion Gap, mEq/L	12.5 [10.0,15.0]	11.5 [9.8,13.5]	12.0 [10.0,14.0]	12.1 [10.3,14.7]	12.0 [11.0,14.0]	<0.001	2.04%
Bicarbonate, mEq/L	23.0 [21.0,26.0]	24.4 [21.9,27.0]	23.0 [20.0,25.0]	22.9 [19.9,25.3]	22.0 [19.0,23.8]	<0.001	2.01%
Blood Urea Nitrogen, mg/dL	23.0 [13.0,48.0]	18.0 [11.0,34.0]	19.0 [13.0,36.0]	21.0 [14.0,44.8]	18.0 [11.0,28.0]	<0.001	2.95%
Calcium, mEq/L	7.9 [7.5,8.3]	8.4 [8.0,8.7]	8.1 [7.8,8.5]	8.1 [7.7,8.5]	8.3 [8.0,8.7]	<0.001	2.33%
Chloride, mEq/L	104.0 [101.0,108.0]	103.0 [100.0,106.0]	104.0 [101.0,108.0]	104.0 [101.0,109.0]	103.0 [100.0,107.0]	<0.001	2.23%
Creatinine, mg/dL	1.2 [0.8,2.1]	0.9 [0.7,1.5]	1.0 [0.8,1.7]	1.0 [0.8,1.7]	0.9 [0.7,1.2]	<0.001	2.06%
Glomerular Filtration Rate, mL/min	52.0 [29.0,74.2]	43.3 [21.8,54.6]	62.0 [34.2,91.0]	39.7 [17.5,53.8]	78.0 [53.0,106.0]	<0.001	27.55%
Glucose, mg/dL	117.0 [95.0,171.0]	112.0 [95.0,152.0]	111.5 [93.0,160.0]	122.0 [99.0,175.5]	104.0 [89.8,140.2]	<0.001	2.04%
Potassium, mEq/L	4.2 [3.8,4.7]	4.0 [3.6,4.5]	4.3 [3.9,4.7]	4.3 [3.9,4.7]	4.3 [4.0,4.7]	<0.001	2.18%
Sodium, mEq/L	140.0 [137.0,143.0]	138.0 [135.0,141.0]	139.0 [136.0,142.0]	140.0 [136.0,143.0]	137.0 [135.0,140.0]	<0.001	2.06%
**Liver Function**							
Albumin, g/dL	3.3 [2.9,3.5]	2.9 [2.5,3.2]	2.8 [2.4,3.1]	2.7 [2.4,3.0]	2.9 [2.6,3.2]	<0.001	6.83%
Alkaline Phosphatase, units/L	68.0 [54.0,91.0]	79.0 [61.0,106.0]	74.0 [58.0,95.0]	82.0 [62.0,110.0]	77.0 [60.2,104.0]	<0.001	6.95%
Alanine Aminotransferase, units/L	27.0 [17.0,49.0]	30.0 [18.0,53.0]	30.0 [18.0,53.0]	35.0 [22.0,61.2]	29.0 [19.0,52.0]	<0.001	6.97%
Aspartate Aminotransferase, units/L	42.0 [27.0,70.2]	41.0 [28.0,65.0]	44.0 [30.0,72.0]	48.0 [30.0,78.2]	43.0 [28.0,71.0]	<0.001	7.17%
Total Bilirubin, mg/dL	0.6 [0.4,0.8]	0.6 [0.4,0.8]	0.6 [0.4,0.8]	0.6 [0.4,0.8]	0.5 [0.4,0.7]	<0.001	8.54%
Total Protein, g/dL	6.5 [6.0,7.0]	6.4 [6.0,6.9]	6.5 [6.1,6.9]	6.4 [5.9,6.8]	6.5 [6.0,7.0]	0.008	6.75%
**Inflammatory Markers**							
C Reactive Protein, mg/L	147.5 [74.4,243.3]	112.7 [54.0,195.2]	138.4 [75.3,218.8]	128.8 [61.1,213.3]	105.1 [46.9,194.9]	<0.001	21.32%
**Hematological Markers**							
Hematocrit, %	38.1 [33.5,42.3]	37.7 [32.8,42.0]	39.8 [35.1,43.2]	37.9 [33.5,42.1]	39.0 [34.6,42.9]	<0.001	2.26%
Hemoglobin, mEq/L	12.3 [10.7,13.6]	12.2 [10.5,13.5]	12.8 [11.1,13.8]	12.7 [11.2,14.1]	12.7 [11.2,13.8]	<0.001	2.46%
Lymphocyte Count	1.1 [0.7,1.5]	0.9 [0.6,1.3]	1.1 [0.8,1.4]	0.9 [0.6,1.3]	1.1 [0.8,1.5]	<0.001	8.79%
Lymphocyte Percentage, %	14.6 [8.4,22.6]	12.9 [7.2,20.4]	13.7 [8.6,21.0]	11.1 [7.0,17.5]	13.9 [8.9,20.4]	<0.001	6.25%
Mean Corpuscular Hemoglobin Concentration, g/dL	29.3 [27.3,30.8]	29.4 [27.9,30.7]	30.0 [28.1,31.2]	30.7 [29.3,32.0]	29.9 [28.4,31.2]	<0.001	2.48%
Mean Corpuscular Volume, fL	91.1 [86.6,95.2]	89.1 [85.4,92.5]	92.9 [88.6,96.9]	92.0 [88.3,95.8]	91.6 [87.5,94.6]	<0.001	2.90%
Monocyte Count	0.5 [0.3,0.7]	0.5 [0.3,0.7]	0.5 [0.3,0.7]	0.5 [0.3,0.7]	0.5 [0.3,0.8]	<0.001	8.71%
Monocyte Percentage, %	6.4 [4.4,9.2]	6.4 [4.0,9.4]	6.3 [4.1,9.0]	6.0 [3.8,8.8]	6.6 [4.4,9.2]	0.133	6.21%
Mean Platelet Volume, fL	8.5 [7.7,9.4]	8.6 [7.9,9.5]	8.6 [7.8,9.5]	8.4 [7.6,9.4]	8.4 [7.7,9.3]	0.001	2.98%
Neutrophil Count	5.5 [3.6,8.8]	5.3 [3.5,8.5]	6.0 [4.1,9.5]	6.5 [4.3,9.8]	6.3 [3.9,9.8]	<0.001	6.23%
Neutrophil Percentage, %	77.4 [67.3,84.6]	78.2 [68.0,86.0]	78.3 [69.0,85.5]	79.4 [69.7,86.0]	77.7 [67.7,84.5]	0.11	6.48%
Platelet Count	207.0 [153.0,270.0]	221.0 [166.5,295.0]	200.0 [151.0,274.0]	228.5 [163.2,310.0]	210.0 [159.0,275.0]	<0.001	2.61%
RBC Count	4.2 [3.6,4.8]	4.2 [3.6,4.6]	4.3 [3.8,4.7]	4.2 [3.6,4.6]	4.3 [3.8,4.7]	<0.001	2.61%
Red Blood Cell Distribution Width, %	13.3 [12.6,14.4]	14.5 [13.7,16.0]	12.8 [12.2,14.0]	12.8 [12.1,14.1]	12.7 [12.0,13.8]	<0.001	3.45%
White Blood Cell Count, 103uL	7.4 [5.3,10.5]	7.0 [5.0,10.2]	7.7 [5.6,10.9]	8.0 [5.8,11.2]	8.0 [5.8,11.3]	<0.001	2.51%
**Mortality within 7 days, n (%)**	148 (24.2)	118 (7.2)	93 (12.4)	124 (23.0)	27 (5.6)	<0.001	0.00%
