# Supplementary material for: Federated Learning of Electronic Health Records to Improve Mortality Prediction in Hospitalized Patients With COVID-19: Machine Learning Approach
Source: JMIR Med Inform. 2021 Jan 27;9(1):e24207. doi: 10.2196/24207 (PMC7842859; doi:10.2196/24207)
Supplement: Multimedia Appendix 3 [file medinform_v9i1e24207_app3.pdf]

Supplementary Table 2: Effects of Class Balancing Techniques on MLP Local AUC-ROC and AUPRC.

		Area under the receiver operating-characterstic (AUC-ROC)				
	Patients	Unbalanced	Static Class Weights (0.05,0.95)	Proportional Class Weights	1:1 Undersampling	% Mortality
Mount Sinai Brooklyn (MSB)	611	0.840	0.852	0.852	0.792	17.45%
Mount Sinai Hospital (MSH)	1644	0.818	0.808	0.808	0.768	4.67%
Mount Sinai Morningside (MSM)	749	0.793	0.811	0.810	0.820	9.98%
Mount Sinai Queens (MSQ)	540	0.776	0.795	0.783	0.789	13.68%
Mount Sinai West (MSW)	485	0.806	0.885	0.892	0.810	5.71%
			Area under the precision-recall Curve (AUPRC)			
	Patients	Unbalanced	Static Class Weights (0.05,0.95)	Proportional Class Weights	1:1 Undersampling	% Mortality
Mount Sinai Brooklyn (MSB)	611	0.647	0.684	0.666	0.585	17.45%
Mount Sinai Hospital (MSH)	1644	0.211	0.196	0.197	0.157	4.67%
Mount Sinai Morningside (MSM)	749	0.387	0.430	0.421	0.444	9.98%
Mount Sinai Queens (MSQ)	540	0.558	0.584	0.562	0.555	13.68%
Mount Sinai West (MSW)	485	0.110	0.163	0.174	0.079	5.71%
