# Supplementary material for: Federated Learning of Electronic Health Records to Improve Mortality Prediction in Hospitalized Patients With COVID-19: Machine Learning Approach
Source: JMIR Med Inform. 2021 Jan 27;9(1):e24207. doi: 10.2196/24207 (PMC7842859; doi:10.2196/24207)
Supplement: Multimedia Appendix 4 [file medinform_v9i1e24207_app4.pdf]

SUPPLEMENTARY TABLE 3: TRIPOD Guidelines Report.

Topic	#	Description	Manuscript Location
Title	1	Federated Learning of Electronic Health Records to Improve Mortality Prediction in Hospitalized Patients With COVID-19: Machine Learning Approach<br>- Identifies the target population (COVID-19 Positive New York City Patients), outcome (Mortality) and the prediction model (Machine Learning)	Title
Abstract	2	Objective - To develop a Machine Learning model for predicting patient mortality within seven days on the basis of admission variables<br>Study design- Utilized Federated MLP Federated LASSO and baseline comparator models along with pooled models. Local models were trained and validated on patients within one hospital. Pooled models were trained and validated on data aggregated from all five hospitals. Federated models were trained independently, and parameters were sent to a central aggregator.<br>Setting- 5 hospitals in the Mount Sinai Health System<br>Participants- New York City confirmed COVID-19 positive patients<br>Sample size- 4029 patients<br>Predictors- demographics, past medical history, lab test results, vital signs<br>Outcome- Predicting in-hospital mortality<br>Statistical analysis- Federated MLP, Federated LASSO, MLP, and LASSO<br>Results- On the training set, both federated MLP and federated LASSO showed performance improvements as measured by area-under the receiver-operating-curve (AUC-ROC) and decrease in loss as models were arbitrarily trained for 80 epochs. Both federated models outperformed their local counterparts at four hospitals. Federated MLP consistently	Abstract

		outperformed federated LASSO at all five hospitals. Federated MLP had higher AUC-ROCs at all five hospitals than pooled MLP models while federated LASSO did not have higher AUC-ROC at any hospital.<br>Conclusion- Federated models were better at predicting outcomes than local models and often better than pooled models.	
Background, Objectives	3a	Medical context- Over 42 million people have tested positive for SARS-CoV-2 worldwide. Patients with COVID-19 demonstrate varying symptomatology, making triaging difficult.<br>Rationale for developing model-<br>Valuable data on predicting clinical outcomes exists around the world but remains siloed within institutions. Federated learning offers an approach to protect patient privacy while utilizing data to develop improved machine learning models to improve patient outcomes.<br>Thus, we developed a federated MLP and federated LASSO model utilizing five hospitals within the Mount Sinai Health System to gauge the effectiveness of federated learning models in a real-life scenario.	Introduction
Background, Objectives	3b	Objective- To predict mortality using key patient characteristics of patients with confirmed COVID-19 status<br>Development of federated learning models trained on EHR from five hospitals and compared to comparable local and pooled machine learning models.	Introduction
Sources of Data	4a	Cohort of all COVID-19+ primary hospitalizations at five hospitals from MSHS, Electronic Health Records, Aggregated by the Mount Sinai COVID Informatics Center.	Results
Sources of Data	4b	March 15, 2020 to May 22, 2020	Results
Participants	5a	Hospitalized patients at 5 NYC hospitals in the Mount Sinai Health System	Results

Participants	5b	Patients >18 years of age with a positive SARS-CoV2 RT-PCR test that was placed within 48 hours of admission and were intubated <48 hours after admission.	Figure 1
Participants	5c	Treatments include full gamut of hospital events, but notably include intubation and ICU admission.	
Outcome	6a	Mortality (death) at 7 days.	Results
Outcome	6b	490 fold bootstrapping where each experiment had a 70-30 training-testing split and was initialized with a unique random seed.	Statistical Analysis
Predictors	7a	Predictors included available patient demographics, medical history, vitals at intake, and labs on admission (within 36 hours). These were developed based on input from a team of front-line clinicians into what was clinically relevant and comprehensive.	Table 1
Predictors	7b	We built machine learning models based on federated LASSO, federated MLP, local LASSO, pooled LASSO, local MLP, and pooled MLP.	Statistical Analysis
Sample Size	8	The sample size differed based on time window and site but in total we had data for 4,029 total patients.	Figure 1, Methods
Missing Data	9	For all models, predictors were removed if missing in >30% of patients and the rest were imputed using K nearest neighbors.	Model development and Selection
Statistical analysis methods	10a	All remaining predictors were used in all models for prediction.	Model development and Selection
Statistical analysis methods	10b	Models used in this study included a federated MLP, federated LASSO, pooled MLP, local MLP, pooled LASSO, and local LASSO.	Model development and Selection
Statistical analysis methods	10c	Models were validated within each hospital using 490 fold bootstrapping where each experiment had a 70-30 training-testing split	Statistical Analysis

		and was initialized with a unique random seed.	
Statistical analysis methods	10d	Measures used to compare models included model accuracy, sensitivity, specificity, AUC-ROC, AUC-PRC, and F1-statistic.	Statistical Analysis
Statistical analysis methods	10e	No model recalibration was performed after training.	
Risk groups	11	No risk groups were created.	
Development vs. validation	12	We developed the federated models by sending parameters to a local site and fitting them on the local data. Weights were then returned to a central aggregator to updated the models.	
Participants	13a	Flow of participants- Mortality was recorded at 7 day intervals after admission.	Figure 1, Table 2, Results
Participants	13b	Demographics, Medical history, Vital signs, Admission laboratory parameters<br>Please see Supplementary Table 1 for missing data for all predictors and outcomes (mortality)	Table 1<br><br>Supplementary Table 2
Model development	14a	We show the number of patients involved and the proportion of events in Table 1.	Table 1
	14b		
Model specification	15a	This does not apply to our model, but see the GitHub repository for model specifications and Supplementary Table 4 for Hyperparameters.	https://github.com/HPIMS/CovidFederatedMortality, Supplementary Table 4
	15b	The prediction model cannot be used on other cohorts directly, per se, but we do release code in order to replicate how to build the model off identical data.	https://github.com/HPIMS/CovidFederatedMortality

Model performance	16	Please refer to paragraph 3 of “Classifier training and performance”	Results, Figure 2, Table 2
Limitations	18	Details on study limitations include missingness present in admission labs, temporal evolution of COVID management and resource constraints, and intrasystem policies affecting care.	Discussion
Interpretation	19a	Please see manuscript for full discussion.	Interpretation, Results, Discussion
	19b	Encouraging results with decent AUC-ROC on federated models that showed evidence of improved results over locally trained models.	Results
Implications	20	Model may have utility in identifying patients who may die within seven days of admission and can optimize resource management at time of admission.	Discussion
Supplementary	21	Information about baseline patient characteristics, final model hyperparameters, model performance.	Main Figures 2-3, Tables 1-2, and Supplementary Tables 1,4,5
Funding	22	This work was supported by U54 TR001433-05, National Center for Advancing Translational Sciences, National Institutes of Health. The funder had no role in the writing of the manuscript or the decision to submit it for publication.	Funding
