# Supplementary material for: Federated Learning of Electronic Health Records to Improve Mortality Prediction in Hospitalized Patients With COVID-19: Machine Learning Approach
Source: JMIR Med Inform. 2021 Jan 27;9(1):e24207. doi: 10.2196/24207 (PMC7842859; doi:10.2196/24207)
Supplement: Multimedia Appendix 5 [file medinform_v9i1e24207_app5.pdf]

Supplementary Table 4: Final Model Hyper-Parameters.

**Hyper-Parameter**	**LASSO Models**	**MLP Models**
Penalty	L1	---
C-value	0.1	---
Hidden Layers and Units	---	Hidden Layer 1: 40 units<br>Hidden Layer 2: 10 units<br>Hidden Layer 3: 2 units<br>Output Layer: 1 unit
Activation Function	---	Rectified Linear Unit (ReLU)
Optimization Function	---	Adam
Loss Function	---	Logarithmic Softmax
Dropout	---	None
Batch Size	---	32
Learning Rate	---	0.001
Epochs per Round	---	80
