# Supplementary material for: Federated Learning of Electronic Health Records to Improve Mortality Prediction in Hospitalized Patients With COVID-19: Machine Learning Approach
Source: JMIR Med Inform. 2021 Jan 27;9(1):e24207. doi: 10.2196/24207 (PMC7842859; doi:10.2196/24207)
Supplement: Multimedia Appendix 6 [file medinform_v9i1e24207_app6.pdf]

Supplementary Table 5: Model Performance Metrics by Site.

		**Mount Sinai Brooklyn (MSB)**					
		**AUROC**	**AUPRC**	**ACC**	**SENS**	**SPEC**	**F1S**
**LASSO**	Local	0.791 (0.788 - 0.795)	0.583 (0.577 - 0.589)	0.749 (0.744 - 0.755)	0.736 (0.732 - 0.741)	0.702 (0.699 - 0.706)	0.584 (0.580 - 0.588)
	Pooled	0.816 (0.814 - 0.819)	0.580 (0.575 - 0.586)	0.723 (0.719 - 0.728)	0.784 (0.780 - 0.788)	0.701 (0.697 - 0.705)	0.605 (0.602 - 0.608)
	Federated	0.793 (0.790 - 0.796)	0.541 (0.536 - 0.546)	0.710 (0.706 - 0.714)	0.755 (0.750 - 0.760)	0.680 (0.676 - 0.684)	0.593 (0.590 - 0.596)
**MLP**	Local	0.822 (0.820 - 0.825)	0.602 (0.596 - 0.607)	0.746 (0.742 - 0.750)	0.804 (0.799 - 0.809)	0.693 (0.690 - 0.696)	0.617 (0.614 - 0.620)
	Pooled	0.823 (0.820 - 0.826)	0.592 (0.587 - 0.597)	0.754 (0.750 - 0.758)	0.819 (0.814 - 0.823)	0.684 (0.680 - 0.687)	0.619 (0.616 - 0.623)
	Federated No Noise	0.829 (0.826 - 0.832)	0.614 (0.609 - 0.619)	0.762 (0.757 - 0.766)	0.798 (0.793 - 0.802)	0.699 (0.696 - 0.702)	0.629 (0.625 - 0.632)
	Federated With Noise	0.815 (0.812 - 0.818)	0.593 (0.587 - 0.598)	0.749 (0.745 - 0.753)	0.769 (0.764 - 0.773)	0.691 (0.688 - 0.695)	0.611 (0.608 - 0.615)
		**Mount Sinai Hospital (MSH)**					
		**AUROC**	**AUPRC**	**ACC**	**SENS**	**SPEC**	**F1S**
**LASSO**	Local	0.693 (0.689 - 0.696)	0.173 (0.170 - 0.176)	0.718 (0.708 - 0.728)	0.609 (0.601 - 0.616)	0.665 (0.660 - 0.670)	0.250 (0.246 - 0.254)
	Pooled	0.791 (0.788 - 0.794)	0.245 (0.240 - 0.249)	0.708 (0.702 - 0.713)	0.753 (0.748 - 0.757)	0.683 (0.680 - 0.686)	0.285 (0.282 - 0.288)
	Federated	0.772 (0.769 - 0.774)	0.212 (0.208 - 0.216)	0.679 (0.672 - 0.685)	0.740 (0.734 - 0.745)	0.659 (0.656 - 0.663)	0.266 (0.263 - 0.269)
**MLP**	Local	0.750 (0.747 - 0.754)	0.215 (0.211 - 0.219)	0.739 (0.731 - 0.747)	0.649 (0.641 - 0.657)	0.694 (0.690 - 0.698)	0.288 (0.283 - 0.292)
	Pooled	0.792 (0.789 - 0.795)	0.251 (0.247 - 0.256)	0.738 (0.731 - 0.745)	0.740 (0.733 - 0.746)	0.683 (0.680 - 0.686)	0.302 (0.298 - 0.307)
	Federated No Noise	0.786 (0.782 - 0.789)	0.256 (0.251 - 0.261)	0.773 (0.767 - 0.779)	0.676 (0.668 - 0.685)	0.713 (0.710 - 0.716)	0.327 (0.322 - 0.332)
	Federated With Noise	0.777 (0.773 - 0.780)	0.245 (0.240 - 0.250)	0.765 (0.759 - 0.771)	0.665 (0.657 - 0.673)	0.707 (0.704 - 0.710)	0.317 (0.312 - 0.321)
		**Mount Sinai Morningside (MSM)**					
		**AUROC**	**AUPRC**	**ACC**	**SENS**	**SPEC**	**F1S**
**LASSO**	Local	0.660 (0.656 - 0.664)	0.214 (0.210 - 0.218)	0.585 (0.574 - 0.595)	0.644 (0.632 - 0.655)	0.576 (0.567 - 0.585)	0.316 (0.313 - 0.320)
	Pooled	0.789 (0.785 - 0.792)	0.331 (0.326 - 0.337)	0.721 (0.714 - 0.728)	0.744 (0.736 - 0.752)	0.672 (0.668 - 0.676)	0.421 (0.416 - 0.426)
	Federated	0.767 (0.764 - 0.771)	0.321 (0.315 - 0.326)	0.710 (0.703 - 0.717)	0.715 (0.707 - 0.723)	0.665 (0.661 - 0.670)	0.402 (0.397 - 0.406)
**MLP**	Local	0.747 (0.743 - 0.751)	0.333 (0.327 - 0.339)	0.732 (0.726 - 0.739)	0.657 (0.648 - 0.666)	0.686 (0.682 - 0.690)	0.407 (0.403 - 0.411)
	Pooled	0.751 (0.747 - 0.755)	0.314 (0.308 - 0.319)	0.704 (0.696 - 0.711)	0.703 (0.695 - 0.711)	0.651 (0.647 - 0.655)	0.395 (0.391 - 0.400)
	Federated No Noise	0.791 (0.788 - 0.795)	0.364 (0.358 - 0.370)	0.751 (0.745 - 0.758)	0.728 (0.719 - 0.737)	0.680 (0.677 - 0.684)	0.445 (0.440 - 0.450)
	Federated With Noise	0.782 (0.778 - 0.786)	0.360 (0.353 - 0.366)	0.738 (0.731 - 0.745)	0.705 (0.696 - 0.713)	0.681 (0.677 - 0.685)	0.431 (0.426 - 0.436)
		**Mount Sinai Queens (MSQ)**					
		**AUROC**	**AUPRC**	**ACC**	**SENS**	**SPEC**	**F1S**
**LASSO**	Local	0.706 (0.702 - 0.710)	0.415 (0.410 - 0.421)	0.644 (0.638 - 0.651)	0.686 (0.680 - 0.693)	0.612 (0.606 - 0.618)	0.494 (0.491 - 0.497)
	Pooled	0.734 (0.730 - 0.737)	0.454 (0.449 - 0.460)	0.688 (0.683 - 0.693)	0.699 (0.694 - 0.705)	0.640 (0.635 - 0.645)	0.523 (0.519 - 0.526)
	Federated	0.694 (0.690 - 0.698)	0.407 (0.401 - 0.412)	0.678 (0.672 - 0.684)	0.645 (0.638 - 0.652)	0.632 (0.626 - 0.638)	0.489 (0.486 - 0.493)
**MLP**	Local	0.791 (0.788 - 0.795)	0.524 (0.518 - 0.530)	0.728 (0.723 - 0.733)	0.779 (0.774 - 0.784)	0.662 (0.658 - 0.665)	0.570 (0.565 - 0.575)
	Pooled	0.783 (0.779 - 0.786)	0.499 (0.493 - 0.504)	0.714 (0.708 - 0.720)	0.786 (0.781 - 0.790)	0.645 (0.641 - 0.649)	0.561 (0.557 - 0.565)
	Federated No Noise	0.809 (0.806 - 0.812)	0.555 (0.549 - 0.561)	0.740 (0.735 - 0.746)	0.788 (0.784 - 0.793)	0.679 (0.676 - 0.683)	0.585 (0.581 - 0.589)
	Federated With Noise	0.799 (0.796 - 0.802)	0.541 (0.535 - 0.547)	0.739 (0.733 - 0.744)	0.763 (0.758 - 0.768)	0.674 (0.670 - 0.677)	0.581 (0.576 - 0.585)
		**Mount Sinai West (MSW)**					
		**AUROC**	**AUPRC**	**ACC**	**SENS**	**SPEC**	**F1S**
**LASSO**	Local	0.482 (0.473 - 0.491)	0.059 (0.057 - 0.061)	0.583 (0.563 - 0.603)	0.757 (0.735 - 0.779)	0.313 (0.284 - 0.342)	0.138 (0.135 - 0.142)
	Pooled	0.829 (0.824 - 0.834)	0.258 (0.248 - 0.267)	0.754 (0.744 - 0.764)	0.805 (0.799 - 0.812)	0.709 (0.703 - 0.715)	0.304 (0.296 - 0.312)
	Federated	0.801 (0.796 - 0.807)	0.252 (0.243 - 0.262)	0.749 (0.736 - 0.762)	0.761 (0.753 - 0.769)	0.700 (0.694 - 0.707)	0.300 (0.291 - 0.309)
**MLP**	Local	0.719 (0.711 - 0.727)	0.173 (0.166 - 0.181)	0.722 (0.709 - 0.734)	0.613 (0.597 - 0.630)	0.679 (0.669 - 0.689)	0.254 (0.246 - 0.261)
	Pooled	0.842 (0.837 - 0.847)	0.263 (0.253 - 0.273)	0.744 (0.735 - 0.753)	0.842 (0.833 - 0.851)	0.699 (0.693 - 0.705)	0.303 (0.296 - 0.311)
	Federated No Noise	0.836 (0.830 - 0.841)	0.276 (0.266 - 0.287)	0.780 (0.772 - 0.789)	0.805 (0.793 - 0.816)	0.702 (0.696 - 0.707)	0.328 (0.319 - 0.336)
	Federated With Noise	0.831 (0.825 - 0.837)	0.272 (0.262 - 0.282)	0.773 (0.764 - 0.782)	0.794 (0.783 - 0.805)	0.697 (0.691 - 0.702)	0.321 (0.312 - 0.329)
