# Supplementary figures and images for: Federated Learning of Electronic Health Records to Improve Mortality Prediction in Hospitalized Patients With COVID-19: Machine Learning Approach
Source: JMIR Med Inform. 2021 Jan 27;9(1):e24207. doi: 10.2196/24207 (PMC7842859; doi:10.2196/24207)

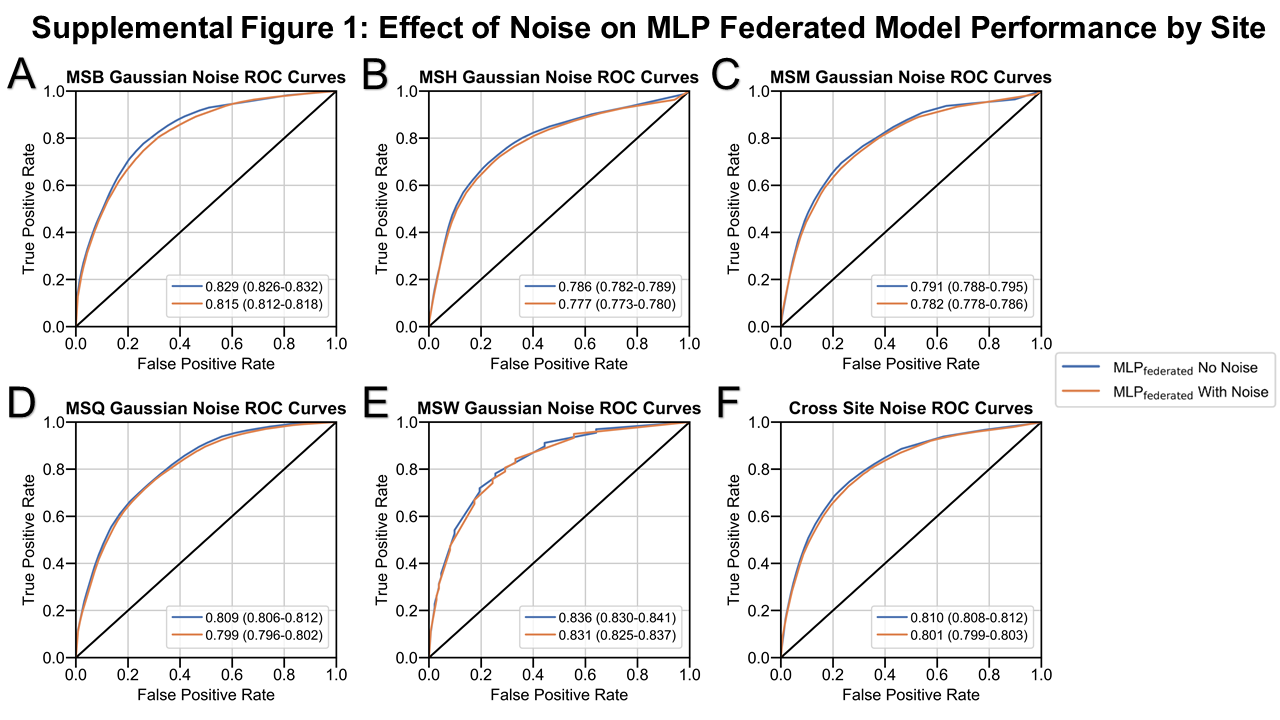

Supplement: Multimedia Appendix 7 [file medinform_v9i1e24207_app7.png]

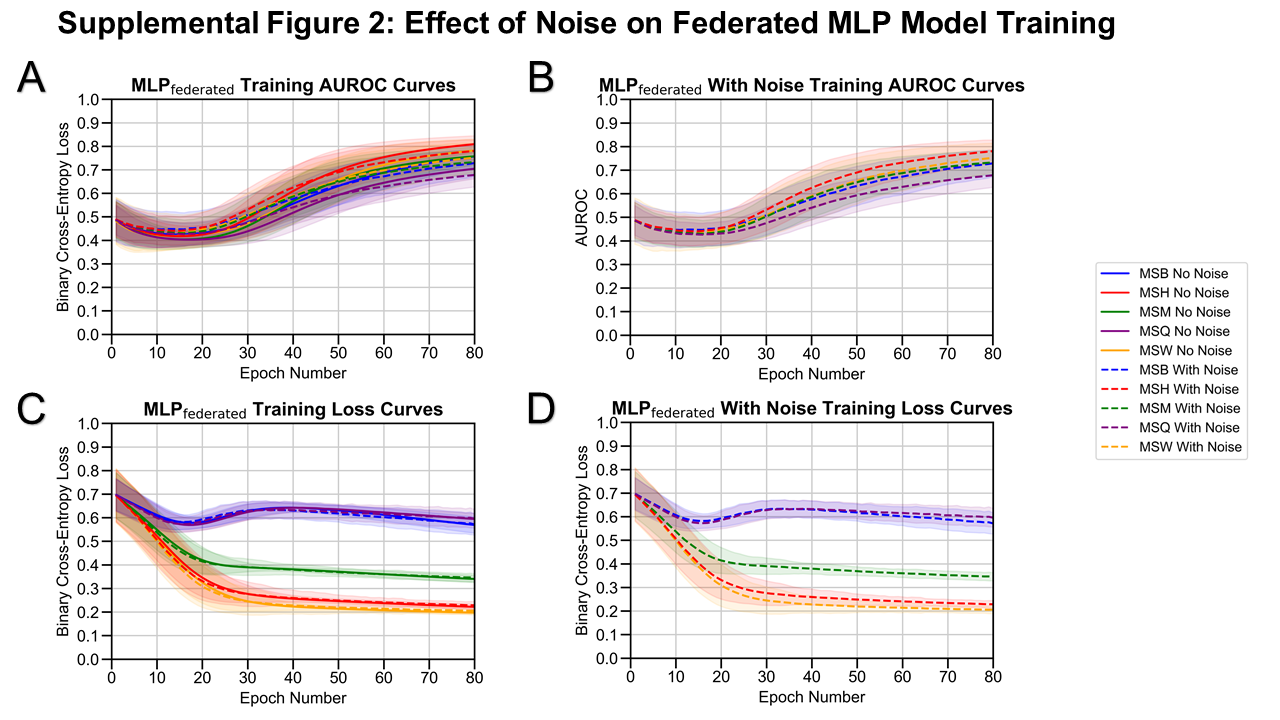

Supplement: Multimedia Appendix 8 [file medinform_v9i1e24207_app8.png]
